# The Burden of HIV Infection among Pregnant Women Attending Antenatal Care in Jimma University Specialized Hospital in Ethiopia: A Retrospective Observational Study

**DOI:** 10.1155/2022/3483767

**Published:** 2022-03-26

**Authors:** Taye Kebede, Michael Dayu, Abiot Girma

**Affiliations:** ^1^Department of Biomedical Sciences and Immunology, Natural Sciences College, Madda Walabu University, P.O. Box 247, Bale-Robe, Ethiopia; ^2^Aklilu Lemma Institute of Pathobiology, Addis Ababa University, P.O. Box 1176, Addis Ababa, Ethiopia; ^3^Department of Internal Medicine, College of Health Sciences and Medicine, Jimma University, P.O. Box 378, Jimma, Ethiopia; ^4^Department of Public Health, College of Health Sciences and Medicine, Jimma University, P.O. Box 378, Jimma, Ethiopia

## Abstract

**Background:**

The HIV (human immunodeficiency virus) epidemic enters its fifth decade amid a global pandemic. Nearly 61% of the people newly infected with HIV live in sub-Saharan Africa (SSA). The virus is transmitted from mother to child during pregnancy, labour, delivery, and breastfeeding, warranting routine counselling at antenatal care (ANC). Hence, this study aimed to determine the prevalence and trend of HIV infection among pregnant women on ANC follow-up at Jimma University Specialized Hospital (JUSH) in Ethiopia from November 2018 to 2021.

**Methods:**

A retrospective cross-sectional study was conducted from June 25, 2021, to November 30, 2021. A total of 634 mothers were sampled by systematic random sampling, and the data were analyzed by descriptive statistics package of SPSS software. A chi-square test was employed to assess an association between variables. Analyses outputs were summarized and presented in tables and figures.

**Results:**

Among the sampled women (634), 96.1% received counselling services on the prevention of maternal-to-child transmission of HIV/AIDS. Around 83.1% of the mothers refused to consult their partners and were unable to persuade their surrogate or afraid to discuss HIV serostatus tests. The overall prevalence of HIV infection among pregnant mothers was 7.1% and no significant decrease in the trends of HIV prevalence over the three years study period (*p* value >0.05). The seroprevalence is high in urban residents (4.4%) and age group of 25–29 years (38.9%) (*p* value <0.05). Residence, level of education attained, and marital status of women were significantly associated (*p* value <0.05) with seropositivity.

**Conclusion:**

HIV burden among ANC attendees in JUSH is high as compared to the national figure and its trend over three years is steady. Accordingly, mandatory early screening tests and community-based education are mandatory for all women and adolescent girls in the reproductive age group.

## 1. Introduction

According to the 2019 global summary figure, 31.6–44.6 million people were living with HIV and 1.2–2.2 million people acquired HIV infection. More than half of the people newly infected with HIV live in SSA. At a look into a decade trend and progress from 2010 to 2019, the HIV/AIDS (acquired immunodeficiency syndrome) epidemic is on the rise in eastern Europe and central Asia, with the number of people acquiring HIV increasing by more than 70%. Similarly, the decade quantitative assessment reveals an increase of more than 20% in North Africa, Latin America, and the Middle East [1].

The United Nations General Assembly agreed that ending AIDS (acquired immunodeficiency syndrome) as a public health threat by 2030 requires a fast-track response, with four milestones to be reached by 2020 [2]. These milestones include reducing the number of people newly infected with HIV to fewer than 500000 per year globally, reducing the number of people dying from AIDS-related causes to fewer than 500000 per year globally and eliminating HIV-related stigma and discrimination [3]. Although the number of people dying from AIDS-related causes has steadily declined by nearly one-third and annual incidence is the lowest since 1989, these global targets have not been achieved and remain a significant challenge [1, 3].

In SSA, more than 50% of rural young women (15–24 years of age) have been pregnant before their 18^th^ year birthday from the two hundred million women and girls living in LMICs countries. The available data from the LMICs reveal that 7 in 10 young women in SSA do not have comprehensive knowledge about HIV. Knowledge about HIV prevention among young people has remained stagnant over the past 2 decades. Globally, only 1 in 4 young people have accurate knowledge about HIV prevention and transmission, as comprehensive sexuality education programs are often limited. It is only 29.8% of young women in SSA have basic knowledge about how to protect themselves from HIV. Specifically, in western and central Africa, both knowledge about HIV and condom use is low among young people (aged 15–24 years), with fewer than one in four (23%) young women having comprehensive and correct knowledge about how to prevent themselves from HIV [4].

The epidemiological rationale for HIV programs focuses on key population groups, as these key populations continue to experience a significant HIV burden, and they influence the dynamics of HIV epidemics. In many African countries, certain groups are particularly vulnerable to HIV infection, for example, migrant workers, refugees, long-distance truck drivers, military personnel, miners, children, youth, youth people, and young women. These populations are not uniformly vulnerable or equally affected across different countries and epidemic settings [5].

The universal health coverage (UHC) set by member countries of the UN is designed to resolve the determinants of infectious diseases, including HIV/AIDS, and its comorbidities through multisectoral actions [6]. The Sustainable Development Goal (SDG) targets are stipulated for 2030, including the empowerment of women and girls, including access to quality essential healthcare services. In September 2019, UN Member States reaffirmed their commitment to this target at a UN General Assembly high-level meeting on UHC. In addition, they set a new target that an additional 1 billion people have access to quality essential health services by 2023 [7]. However, today, nearly half of the people living with HIV/AIDS are females. Sadly, even more ominously, in SSA, female individuals infected with HIV are around 60%, where 14 infected women for every 10 infected men [8]. This number rises to 66% in the African women of the age group 15–24 years, the most productive age group of the society [9].

Almost 4 in 5 people living with HIV globally knew their serostatus in 2018, and two-thirds of all people living with HIV in 2018 are receiving life-saving antiretroviral therapy (ART), and more than half of them are capable to suppress their viral loads. An estimated 23.3 million of the 37.9 million people living with HIV globally were on treatment, more than four times as many as in 2010 [10]. But, in contrast to this success story, the rise of resources cost for HIV responses in LMICs stalled in 2017 and funding decreased by 7% between 2017 and 2019. This collective failure to invest sufficiently in comprehensive, rights-based HIV responses comes at a terrible price throughout 2015–2020, where there were 3.5 million more HIV infections and 820000 more AIDS-related deaths than if the world was on track to meet its 2020 targets [11].

Couples' voluntary HIV counselling and testing (CVCT) is a high-impact HIV prevention intervention in Rwanda and Zambia. Also, CVCT averts 54% (in Sierra Leone) and 62% (in South Africa) of adult HIV infections. Especially, a nationalized CVCT prevents over half of adult HIV infections [12]. Similarly, in a study conducted over two years period in Abidjan, voluntary counselling and HIV testing to prevent mother-to-child transmission was feasible in antenatal care units and was well accepted by pregnant women. But, many HIV-positive women discontinue ANC follow-up, the primary target group for preventing mother-to-child transmission of HIV [13]. For the wider scale and successful voluntary tests, improving women knowledge, well-functioning health system, and preventing home delivery have crucial roles [14].

Nearly a decade ago, a survey conducted in Ethiopia estimated an overall adult HIV prevalence of 1.5%. Genderwise, the prevalence in women was 2%, while that of men was 1%. This same survey reported that the prevalence of HIV among women aged 15–49 years was 1.9%. Spatially, the prevalence was 4.2% in urban dwellers and 0.6% in rural. In terms of regional burden, HIV prevalence was highest in Gambella (6.5%), followed by Addis Ababa administration city (5.2%). Moreover, the survey analysis in terms of educational status revealed that the prevalence was higher (3.1%) in the middle-level academic background (attended secondary school) than less or more level. Furthermore, individuals (both sex) in the highest wealth quintile bear the highest HIV prevalence load (3.9%) [15].

Despite the increased number of pregnant mothers having their ANC follow-up at JUSH, there is limited research work showing the prevalence of HIV among pregnant mothers on ANC follow-up at JUSH. Hence, this study aimed to highlight the burden and trend of HIV infection among pregnant women on follow-up.

## 2. Materials and Methods

### 2.1. Study Area and Period

The study was conducted at JUSH, southwest Ethiopia, Jimma city, from June 25, 2021, to November 30, 2021. Jimma city is found in Jimma zone, Oromia administrative regional state, and is the capital of Jimma zone and located at about 346 km in the southwest direction of the country's capital city, Addis Ababa. As per the 2007 population census's projection for the year 2017, Jimma Zone has an estimated population of 3,261,371. Of the total population, 49.9% were women. Among the females, 23.1% were women of the childbearing age, 753377 women. Jimma Zone has only 1 administration city (Jimma city), 20 rural districts, and 1 special town administration (Agaro town), 46 rural towns, and 512 rural kebeles (the smallest hierarchical demographic units) [16]. The Jimma city has a population of 120960 (60824 male and 60136 female) and 32191 households. It is subdivided into two districts and 13 kebeles. Jimma administration city gets an annual rainfall of 3700 mm and is located at an altitude height range of 1500–17000 above the sea level. Currently, there is only one specialized teaching hospital, JUSH in Jimma city [17].

### 2.2. Study Design

A retrospective cross-sectional study design was employed in the current study.

### 2.3. Source Population and Study Population

The source population of this study was all pregnant mothers who visited the ANC unit of JUSH between 2018 and 2021. Whereas, the study population of this study was all pregnant women who visited the ANC unit of JUSH from 2018 to 2021 and those who fulfilled the eligibility criteria of this study.

### 2.4. Sample Size Determination

The sample size was determined by single population proportion formula for prevalence study as follows, using *p* of 1.9% [15] and *d* = margin of error = ½ (1.9%) = 0.95%.

(1) $$\begin{array}{c} n=\frac{1}{d^{2}}{\left(\;Z_{1-\alpha /2}\right)}^{2}P\left(1-P\right), \end{array}$$

where *P* represents the prevalence of HIV in pregnant mothers, *d* represents the margin of error, Z_1−*α*/2_ represents the level of significance, and *ո* represents the calculated sample size. Using the above formula, the calculated sample size was 793. Since the source population was less than 10000, which was 3314, the sample size was corrected by using the formula for the finite population.

(2) $$\begin{array}{c} nf=\frac{n}{1+\left(n/N\right)},\\ =\frac{793}{1+\left(793/3314\right)}=634. \end{array}$$

The sampling fraction was 0.19 (634/3314) and sample sizes from 2018 to 2019, from 2019 to 2020, and from 2020 to 2021 were 205, 169, and 260, respectively.

### 2.5. Sampling Technique and Variables of the Study

The sample units were selected by the systematic random sampling technique by reviewing the clients' record archives of ANC follow-up, a logbook for each year, and by calculating *k* value for each year as 5, after the first client was selected by the lottery sampling method. The dependent variable is HIV/AIDS seropositivity status. Whereas, the independent variables assessed were age, educational status, gravidity, marital status, place of residence, duration of the year on ANC follow-up (period), and the client's partner-related factors.

### 2.6. Operational Definitions

AIDS is a disease of the human immune system, which causes the immune system to weaken right off infection, and occurs when CD4 cell count falls below 200 cell/mm^3^ making the person vulnerable to opportunistic infections and AIDS-defining conditions.

ANC is the healthcare given to a pregnant woman to ensure the birth of a healthy baby with minimal health risk to the mother.

HIV is a virus that gradually attacks immune system cells and finally causes AIDS.

HIV infection is the mere presence of HIV antibodies in blood as is detected by an HIV test kit.

Seroprevalence is the number of persons in a population who is positive for a specific disease based on a serological specimen (blood serum).

Gravidity is the total number of pregnancies, either normal or abnormal.

Parity is the state of having given birth to an infant or infants weighing 500 g or more, which might be alive or dead.

### 2.7. Data Collection Process and Technique

Data about maternal characteristics, PMTCT, client's partner-related factors, and maternal HIV seropositivity status were extracted from the client's card by using a checklist prepared for this purpose. Data were exhaustively collected by reviewing the ANC records using a record checklist. Three final year students from the biomedical sciences background were selected to collect the data after they had been trained on data collection tools and basic principles of biomedical research ethics for one day. Also, throughout the study period, all the researchers were fully engaged with the data collection monitoring, regarding the recording and filling of the data on a predesigned checklist by the selected data collectors.

### 2.8. Data Quality Management

Over the data collection period, the checklist was checked for consistency and completeness on an everyday basis at the end of the session by the three data collectors. The results recorded were handled appropriately and stored in a secure place until transcribed into computer applications designed for data entry and statistical software. Moreover, to assure the quality of the questionnaires data on sociodemographic and maternal-associated clinical data, the laboratory test result records were supervised by one of the researchers. The collected data were regularly checked for any error, and the necessary corrections were made on daily basis. Finally, regular and closer supervision was maintained during the whole data collection phase by engaging all the researchers.

### 2.9. Data Analysis

The collected data were checked for completeness at the end of each day data collection session and entered into an Excel spreadsheet overnight. The data analysis was carried out by importing the Excel spreadsheet entered data into SPSS software Window version 22. Descriptive statistical packages of the software were applied together with the chi-square test to assess an association between the dependent and independent variables. To assess the significance level of the variables' association, the *p* value was employed at the level of less than 0.05 (*p* value < 0.05) at a 95% confidence interval. Finally, the analyzed data were presented using numerical and graphic summaries.

## 3. Results and Discussion

### 3.1. Results

#### 3.1.1. Seroprevalence and Sociodemographic Characteristics

**Figure 1:**
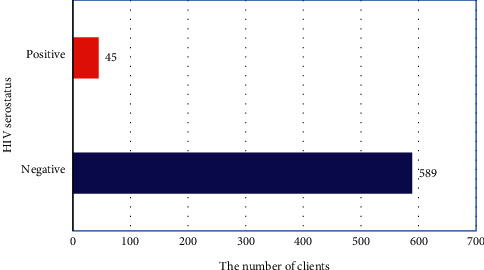
The overall HIV serostatus among pregnant mothers on ANC follow-up at JUSH.

A total of 634 detailed records of ANC attendant pregnant mothers at JUSH were sampled and included in the final analysis. Among those pregnant women selected for the study, the overall prevalence of HIV infection among pregnant mothers was found to be 7.1% (Figure 1). Theoretically, all pregnant mothers who visited the JUSH ANC unit to sought ANC follow-up were assumed tested for prenatal HIV infection. The majority of the pregnant mothers were found in the age interval of the highly productive age. Out of the 415 (65.4%) pregnant mothers who can read and write, around 38 (6%) were HIV seroreactive.

The educational status of HIV-seropositive pregnant women who attended formal education was 4.2%. Around 88.9% of the sampled pregnant mothers were married, while the rest proportion was either widowed or divorced. More than half (63.1%) of the pregnant mothers who attended the ANC unit of JUSH for follow-up came from urban settings. The share of the urban dwellers towards HIV seropositivity was 4.4%. Of all the study participants who were screened for HIV infection, the majority of HIV-seropositive pregnant mothers were in the age group of 25–29 years (46.7%), married (73.3%), and 2.7% attended 1–4 grades schooling in terms of their educational background (Table 1).

#### 3.1.2. HIV Seroprevalence by Maternal Obstetric Characteristics and Male Partners Factors

The majority of the pregnant mothers (49.2%) were multigravida (2–5) with a seroprevalence level of 3.5%. Around 377 (59.4%) pregnant women had started their ANC follow-up before the 28^th^ week where the highest percent (4.2%) of women diagnosed with HIV-seropositive before the age of viability. Almost all (96.1%) of the pregnant mothers were counselled on the prevention of maternal-to-child transmission of HIV/AIDS (Table 2).

**Figure 2:**
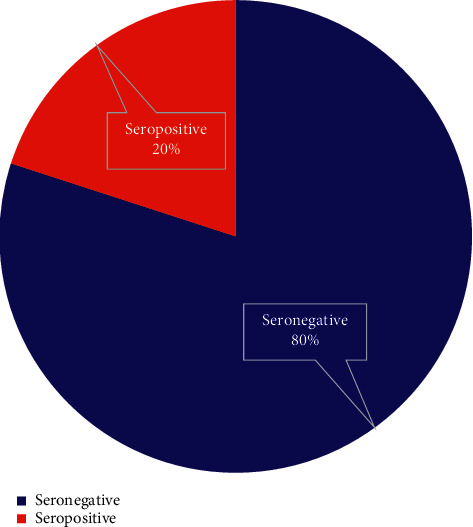
The proportion of tested male partners seropositivity status at JUSH (*N* = 95).

Of those pregnant mothers who attended ANC follow-up at JUSH in southwestern Ethiopia, only 95 (14.9%) of their partners became a volunteer and gave consent to receive HIV infection tests. Among those 95 tested for HIV serostatus, around 20% of them was proven seropositive (Figure 2).

**Figure 3:**
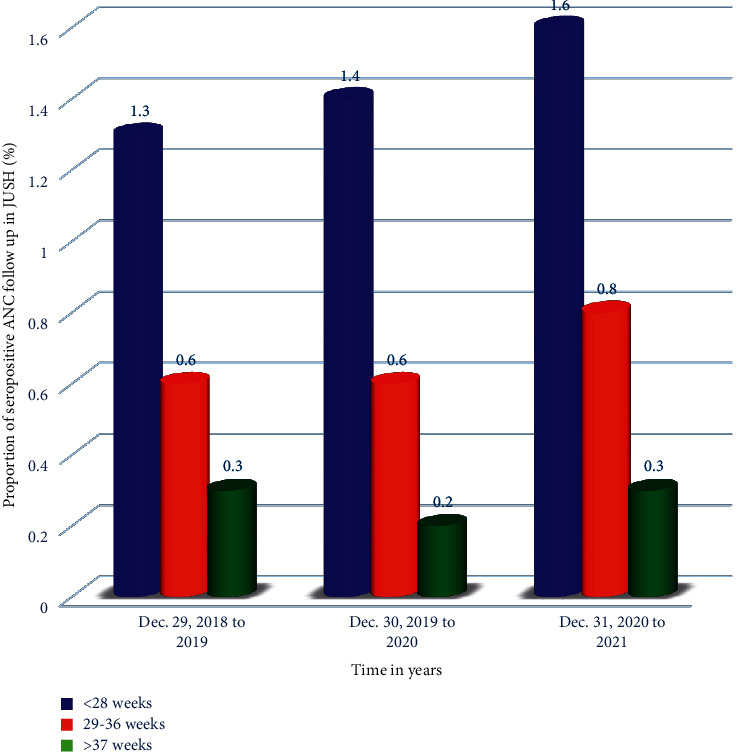
The seropositivity of HIV by gestational weeks and year of study among pregnant mothers on ANC follow-up at JUSH.

Of the antepartum mothers on their ANC follow-up at JUSH from 2018 to 2021, the majority of the seropositive mothers are diagnosed at the gestational weeks before 28 weeks. One-year cumulative seropositivity or prevalence of HIV/AIDS before 28 weeks of the gestational period of women in 2019, 2020, and 2021 years was 1.3%, 1.4%, and 1.6%, respectively (Figure 3).

There is a significant association between the dependent variables (HIV serostatus among the pregnant women on ANC follow-up at JUSH) and the independent variables (maternal-related factors of pregnant women on ANC follow-up at JUSH, the level of education attained by mothers, residence area, and their marital status at 5% level of significance and 95% of confidence interval) (Table 3).

**Figure 4:**
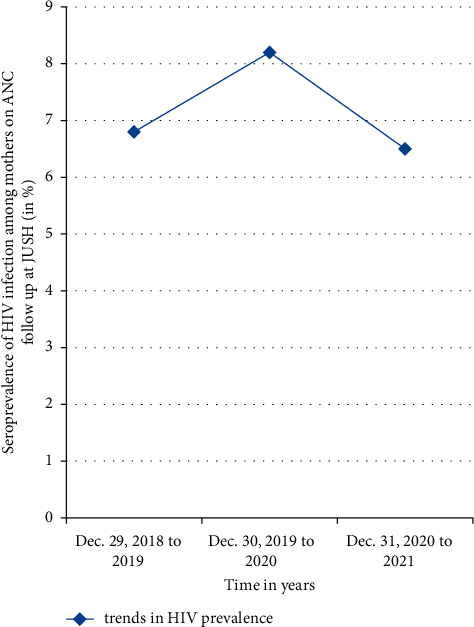
Trend of HIV seropositivity over the consecutive three years among pregnant mothers on ANC follow-up at JUSH.

Despite a slight decrease in the number of pregnant women who visited the ANC parlour in Jimma University Specialized Hospital in 2020 because of the COVID-19 pandemic frustration, the HIV seroprevalence was high. The HIV seroprevalence was high in 2020 (8.2%) as compared to 2019 and 2021 years where the seroprevalence was falling between 6% and 7% (Figure 4).

## 4. Discussion

Ethiopia, like most countries in SSA, is experiencing a high prevalence of HIV with about 2.1% of the adult population living with virus, largely due to heterosexual transmission. Besides heterosexual transmission, vertical transmission of HIV from mother to child accounts for more than 90% of pediatric HIV/AIDS [18]. A few years back, around 53% of HIV-infected pregnant women worldwide received antiretroviral (ARV) drugs to prevent mother-to-child transmission [19]. Previously, the coverage seemed to improve in SSA ranging from 8% to 54% [20] before it has been stalled by the emergence of COVID-19 disease [21]. These PMTCT programs in the African continent are still plagued by multiple other problems [20].

Data from 146 countries show that some have achieved declines in new HIV infections among adults of 50% or more over the last 10 years, while many others have not made measurable progress, and yet others have experienced worrying increases in new HIV infections. Hence, efforts to reach fewer than 500000 new HIV infections by 2020 are off track. The preventative effect of antiretroviral therapy has been limited because 40% (35–44%) of people living with HIV do not know their HIV status and 62% (59 to 65%) of people living with HIV are not virally suppressed; well shy of the 90-90-90 target. Reaching the third 90, which translates to 73% of people living with HIV virally suppressed, can only achieve up to 50% of the incidence reduction required to end the AIDS epidemic by 2030. In the past, present, and far into the future, primary prevention is an essential component of the response [22]. The low utilization of PMTCT needs earnest attention and interventions, as exemplified by expanding seropositivity tests.

In this study, among the total number of 634 women sampled and included, the overall prevalence of HIV infection among the pregnant mothers who were on the ANC visit at JUSH was 7.1%. During the current study period, all volunteer pregnant mothers coming to the JUSH ANC parlour were tested, but we sampled them based on the methodology of our study (design, sampling method, sampling technique, and inclusion criteria). Of those 634, the majority of the mothers (38.9%) were in the age range of 25–29 years, the age group where the highest seropositivity (3.3%) was found. A previous modelling study conducted in Ethiopia [19] predicted that Ethiopia could achieve universal ANC coverage by 2015, where the prevalence of HIV at PMTCT sites could decrease by four-fold. The possible justification for the discrepancy between the current study and the previous study is the difference between the actual figure on the ground and the ambitious assumption made by modelling software.

In another previous study conducted in northwest Ethiopia [23], the seroprevalence of HIV was found almost double of the current study result (13.4%) in the women aged 25–30 years. This reported result from northwest Ethiopia has with the current study, as both studies reflect actual health institution-based tests conducted at ANC clinics. In a more refined dissimilarity, the slightly lower prevalence in the current study could be due to the large sample size (634 pregnant women) in the current study than the previous study (300 pregnant women). Furthermore, a previous mixed-methods study in four African countries [24] reported that mother-to-child transmission of HIV was high in developing nations, particularly SSA countries where the majority of HIV-infected women of childbearing age live. Such high rates persist mostly due to a lack of access to health facilities, awareness, and health education.

Alarmingly, 2 in 5 children living with HIV worldwide do not know their status, and just over half of children with HIV are receiving ART. Some barriers to adequate access to HIV services are longstanding and familiar, including discrimination and gender inequalities. The report notes that many countries saw significant disruptions in HIV services due to COVID-19 in early 2020. HIV infant testing in high burden countries declined by 50–70%, with new treatment initiations for children under 14 years of age falling by 25–50%. Lockdowns contributed to increased infection rates due to spikes in gender-based violence, limited access to follow-up care, and stockouts of key commodities. Several countries also experienced substantial reductions in health facility deliveries, maternal HIV testing, and ART treatment initiation. In an extreme example, ART coverage among pregnant women dropped drastically in some regions significantly in 2020, from 71% to 56% [25].

In this study, in terms of the study participants educational status, out of 415 (65.4%) mothers who can read and write, around 38 (6%) were found HIV-seropositive. Among the women who attended grades 1–4, their collective contribution to HIV seropositivity was 2.7%. A study conducted in 2021 on HIV serostatus among pregnant women in Nekemte public health facilities found that higher educational status was an independent determinant of HIV status in the women sampled [26]. In another previous study [27], one of the women's characteristics that were significantly associated with higher odds of HIV testing results was the educational level (primary and secondary levels). The diminishing sensitivity towards HIV/AIDS prevention in literate African women in this and the indicated two previous studies from Africa might implicate the homogeneity of the sociocultural and religious fidelity in less educated women across the continent.

On the contrary, a study from Zimbabwe [28] refutes the analogy of the rise in HIV serostatus positivity of mothers with an increase in the literacy level of women. The highest level of education attained had a significant association with women's HIV seronegative status, meaning HIV infection decreased with increased educational attainment by women, where women with secondary or tertiary education levels were less likely to be HIV positive compared to women who completed primary education only. The differences between the current study and the Zimbabwean study in 2019 might be due to the sampled women, which was all from the urban area in the case of Zimbabwean postpartum mothers. The Zimbabwean study is in line with a report from Nigeria in 2016 [29], which could be due to the sample unit inclusion of the higher proportion of women from the better educational level background.

In the current study, the highest proportion of the women was married, which is in agreement with a study from Ethiopia in Gondar that reported the majority of women (94%) who were found seropositive for HIV tests were married [23]. In this previous report from Ethiopia, all HIV-seropositive pregnant women were married ones. The reason could be the similarity in sociocultural features and age overlap between the two studies. In this study, the majority of pregnant mothers (88.9%) are married and the seroprevalence was high (5.2%) in this particular group of mothers. In a previous Nigerian study report [29], unlike this study report, HIV prevalence increased consistently with increasing age in the never-married women category and formerly married women (aged 20–24 years) showed the highest prevalence (10%). But no comparison was made by computing straightforward between the married and unmarried categories of women. Again, according to the Zimbabwean study [28], like that of the Nigerian study [29], marriage was protective against HIV infection, as married women were two times less likely to be HIV positive compared to unmarried women.

More than half (63.1%) of the mothers coming to the ANC unit of Jimma University Specialized Hospital for follow-up came from urban areas, among which 4.4% of them were proved HIV seroreactive. The previous study from northwest Ethiopia [23] also reported around 80% of pregnant women who participated in the HIV serostatus study were urban residents. Concerning gravidity, the majority of the mothers (49.2%) were multigravida (in the ranges of 2–5), and it was this gravidity category that leads to the seropositivity (3.5%) of women. The Gondar study reported 13 women (42%) were secundigravidae among the total 31 HIV-seropositive pregnant women. The differences mainly lie in the sample size variation between the current study and that of the report from Gondar.

In our study, about 377 (59.4%) had started their ANC follow-up before 28 weeks (7 months) with the highest percent (4.2%) diagnosed with seropositive before the age of viability. The previous result from Gondar in Ethiopia [23] reported that gestational age of pregnant women who became seropositive was 3.2% in the first trimester, 42% in the second trimester, and a significantly higher proportion (54.8%) in the 7th month.

In the present study, almost all (96.1%) pregnant mothers got counselling services on the prevention of maternal-to-child transmission of HIV. Among the mothers who were on ANC follow-up at JUSH, it was only 95 mothers (14.9%) who practically convinced their partner to get tested for HIV serostatus. Of those 95 male partners who underwent HIV serostatus tests, around 20% of them were found seropositive. A previous research result study [23] indicates that low husband educational status is a risk factor for HIV serostatus. High seroprevalence of HIV was found in women whose husbands attended primary school (19.7%). In another previous research result study in Tanzania from 2003 to 2012 [27], the coefficient of HIV testing uptake increases among ANC attending pregnant women by 44%, being in the age category of 20–24 years by 35%, having primary education by 14% and being a rural resident by 36%.

There is no significant decrease in the trends of HIV seropositivity over the three years study period in this study among the pregnant mothers on follow-up in JUSH (Figure 4). The lack of significant decline in HIV seroprevalence in this study can be explained by the lack or low access to awareness creation campaigns, PMTCT, and ART services. Again, these gaps are compounded by the lack of significant involvement of men in PMTCT services of the family. Despite decrement in the number of ANC follow-up attendant women in 2020, especially the last two gestational trimesters, the HIV seropositivity was the highest (8.2%) as compared to the preceding or following year. In all three years, the seroprevalence is higher than 6.5%; there is a clear indication that the prevention of mother-to-child transmission of HIV-1 continues to be a major public health challenge in southwestern Ethiopia.

This study was conducted on a large cohort of retrospective evaluation of ANC follow-up data from the routine pregnant mothers' visit program in a large specialized hospital in southwestern Ethiopia. Around 71 women among every 1000 ANC attendee women are living with HIV/AIDS and need all the necessary precautions to save their lives and their newborn babies by employing all the necessary assistance recommended by the WHO. The well-being of those HIV-positive mothers and their prospective neonates will be improved through effective and efficient utilization of the highly active antiretroviral therapy (HAART) for pregnant women, elective caesarean sections, and avoidance of breastfeeding [30].

In Ethiopia, the proportion of women of reproductive age who have access to at least one healthcare provider seeking antenatal care, delivery, and/or postnatal care decreased from 96% in 2005 to 70% in 2016 [31]. In the current study, the cumulative seroprevalence of HIV infection among pregnant mothers on ANC follow-up in JUSH (7.1%) is relatively higher than the national data (which is 5.3%) and by far exceeds that of the Oromia administrative regional state, the region in which JUSH found (which is 1.3%) [32]. However, this study finding is lower than the previous prevalence report of HIV infection in pregnant mothers (9.5%) in Amhara administrative regional state in Ethiopia [33] and South Africa (above 20%) [34]. Women are particularly susceptible to HIV infection for both biological and sociocultural reasons like the use of alcohol or chewing the psychotropic plant (Chat) during sexual intercourse, history of multiple sexual partners, and low awareness about the use of condoms during sexual intercourse, transactional sex, inefficient coverage of testing, poor HIV counselling, and testing during pregnancy and low habit of sharing the test results among couples [34].

Africa is inhabited by just over 14.7% of the world's population, but shouldering more than 90% of all HIV/AIDS-related mortality [35]. In Ethiopia, the administrative regions of Oromia (where the current study area is situated), Amhara, Addis Ababa Administration City Council, and Southern Nations, Nationality and People Region (SNNPR), accounted for 86.7% of the total estimated HIV-positive pregnancies and 88.2% of AIDS deaths around a decade and a half ago [31, 32]. By cascading from the global plan, Ethiopia set a goal of universal access and raised the capacity for the delivery of HIV counselling and testing, PMTCT, and provision of ARVs [36]. Despite those plans and some attempts, the number of women accessing these services is still low in regional areas and zones. In addition, health system factors and individual and sociocultural factors have been highlighted as barriers [37]. Moreover, there is an absence of integrated health-related education for successful HAART medication care adherence [38]. Furthermore, the seeming expansion of sex tourism or transactional sex enables the exchange of money, favours, or gifts for sexual intercourse, as such sexual intercourse is associated with a greater risk of contracting HIV [39], because of compromised power relations [40] and the likelihood of having multiple partners [41].

In summary, the prevalence of HIV infection in pregnant mothers is high in southwestern Ethiopia, as women are particularly susceptible to HIV infection for both biological and sociocultural reasons. To curb this devastation, the United Nations member countries set a milestone plan through the Sustainable Development Goal 3.3, which aims to end the epidemics of AIDS by the end of the year 2030. Two-thirds of the estimated 6000 new infections that occur globally each day occur in SSA. In Ethiopia, women in the reproductive ages constitute 23% of the population, but the HIV counselling and testing uptake among Ethiopian youths are still very low. For instance, the number of people living with HIV in Ethiopia was 710000–690000 in 2018, of which 23000 people were newly infected at the end of 2018. ANC follow-up was significantly associated with HIV voluntary counselling and testing. Though unthinkable because of myriads of challenges and bottlenecking issues, breathtaking efforts are going to bring a glimmer of hope for Ethiopia to achieve the milestone set for 2030. Thus, significant measures have to be made regarding prevention, control, and treatment as well as a traumatic social stigma on people living with HIV/AIDS based on actual data from health institutions serving the larger rural communities.

Sadly, about 15.4 million children lost one or both parents to AIDS-related causes last year. Three-quarters of these children, 11.5 million, live in sub-Saharan Africa. Children orphaned due to AIDS make up 10% of all orphans worldwide, but 35% of all orphans live in sub-Saharan Africa. Building back better in a postpandemic world must include HIV responses that are evidence-based, people-centred, resilient, sustainable, and above all, equitable. To close the gaps, these initiatives must be delivered through a reinforced healthcare system and meaningful engagement of all affected communities, especially the most vulnerable [25].

Among the limitations of this study is the use of nonelectronically documented data from the ANC follow-up clinic of JUSH, as it was difficult to control for inconsistencies and missing values. In addition, valuable anthropometric and clinical measurements were excluded from the study simply because of missing (incomplete) values for some study units, as more than 5% missing values out of the total sample size calculated is intolerable. The other limitation is the HIV serostatus evaluation made only in the prepartum period that fails to incorporate the postpartum periods. The fact that all potential factors in women were not included and assessed may somehow affect the generalization of independent predictors in this study. Furthermore, the pregnant women's male partners role in the serodiagnosis and seropositivity were not explored in detail. Despite these limitations, this study sufficiently implicates areas of HIV/AIDS control interventions in the wider rural communities of Ethiopia such as Jimma and revealed the burden of another viral curse (HIV) hampering the welfare of women and their prospective children being shadowed by the current mess of global pandemic, COVID-19 disease.

## 5. Conclusions

Though HIV/AIDS is an easily preventable disease, the burden in southwestern Ethiopia among pregnant women is high and needs urgent attention from the concerned stakeholder bodies. The coverage and quality of prevention of HIV/AIDS services among pregnant mothers in southwestern Ethiopia are questionable and remained persistently low. Targeting pregnant women attending antenatal clinics provides a unique opportunity for implementing prevention of mother-to-child transmission (PMTCT) programs against HIV infection of newborn babies. Hence, Ethiopia has to redesign additional fine-tuning control mechanisms and tailor services of HIV/AIDS prevention accordingly. Besides, to significantly decrease HIV seroprevalence and curb the steady incidence of HIV infection among pregnant mothers, the PMTCT program currently underway in almost all government health facilities needs to be further strengthened both quantitatively and qualitatively. Furthermore, expanding awareness creation of PMTCT campaigns to secondary schools and youth centres has paramount importance in HIV infection prevention and control opportunities.

## Figures and Tables

**Table 1:** HIV serostatus by maternal sociodemographic characteristics among pregnant mothers on ANC follow-up at JUSH.

Variables		Serostatus of the mothers			
		Positive (*n* = 45)		Negative (*n* = 589)	
		Count	%	Count	%
Maternal age	15–19 years	2	4.4	76	12.9
	20–24 years	13	28.9	170	28.9
	25–29 years	21	46.7	226	38.4
	30–34 years	5	11.1	88	14.9
	35–39 years	3	6.7	26	4.4
	40–44 years	1	2.2	3	0.5

Marital status	Married	33	73.3	531	90.1
	Widowed	7	15.6	47	8
	Divorced	5	11.1	11	1.9

Residence	Urban	28	62.2	372	63.2
	Rural	17	37.8	217	36.8

Educational status©	Cannot read and write	7	15.5	212	36
	Read and write only	12	26.7	110	18.7
	1–4	17	37.8	96	16.3
	5–8	5	11.1	97	16.5
	9–12	1	2.2	62	10.5
	Higher education	3	6.7	12	2

Educational status©, **e**ducational status of mothers.

**Table 2:** HIV seroprevalence by maternal obstetric characteristics and male partner-related factors among pregnant mothers on ANC follow-up at JUSH.

Maternal-related factor		Serostatus of the pregnant mothers at JUSH					
		Seropositive		Seronegative		Total	
		Count	%	Count	%	Count	%
Gravidity	1	17	2.7	101	15.9	118	18.6
	2–5	22	3.5	290	45.7	312	49.2
	>5	6	0.9	198	31.2	204	32.2

Gestational age	<28 weeks	27	4.2	350	55.2	377	59.4
	28–36 weeks	13	2.1	194	30.6	207	32.6
	>37 weeks	5	0.7	45	7.1	50	7.8

PMTCT-related counselling	Counselled	43	6.7	566	89.4	609	96.1
	Not counselled	2	0.3	23	3.6	25	3.9

**Table 3:** Factors associated with maternal HIV serostatus among pregnant mothers on ANC follow-up at JUSH (*N* = 634).

Variables		Mothers' HIV serostatus		*χ* ^2^	*P* value
		Positive	Negative		
		Frequency	Frequency		
Marital status	Married	33	531	18.2	<0.05
	Widowed	7	47		
	Divorced	5	11		

Residence	Urban	28	372	0.016	<0.05
	Rural	17	217		

Educational status©	Not read and write	7	212	24.7	<0.05
	Read and write only	12	110		
	1–4 grades	17	96		
	5–8 grades	5	97		
	9–12 grades	1	62		
	Higher education	3	12		

Educational status©, educational status of mothers.

## Data Availability

The datasets used and/or analyzed during the current study are available from the corresponding author upon request.

## Abbreviations

AIDS: Acquired immune deficiency syndrome
ANC: Antenatal care
ART: Antiretroviral therapy
CBE: Community-based education
FMOH: Federal Ministry of Health
HIV: Human immunodeficiency virus
IGO: Institute of Gynecology and Obstetrics
IRB: Institutional review board
JU: Jimma University
JUSH: Jimma University Specialized Hospital
MWU: Madda Walabu University
PMTCT: Prevention of maternal-to-child transmission
SNNPR: Southern Nations, Nationalities and Peoples Region
UNAIDS: United Nations Program on AIDS
VCT: Voluntary counselling and testing
WHO: World Health Organization.
